# Incidence and Genetic Investigation of Avian Coronaviruses in Migratory Ducks From South Korea

**DOI:** 10.1155/2024/9502737

**Published:** 2024-11-01

**Authors:** Yunhee Gim, Song Hwi Jeong, Young Ju Lee, Guehwan Jang, Changhee Lee

**Affiliations:** ^1^College of Veterinary Medicine and Virus Vaccine Research Center, Gyeongsang National University, Jinju 52828, Republic of Korea; ^2^College of Veterinary Medicine and Zoonoses Research Institute, Kyungpook National University, Daegu 41566, Republic of Korea

## Abstract

Coronaviruses (CoVs) belonging to the *Gamma-CoV* and *Delta-CoV* genera are widespread in poultry and wildfowl. Migratory birds, particularly duck species, serve as hosts for CoVs and play a pivotal role in transmitting the viruses to other species, including mammals. Despite the potential risks to animals and humans, there remains a narrow knowledge of the genetic and epidemiological properties of CoVs in wild birds. The current research aimed to detect and characterize CoVs present in migratory duck species (*Anas acuta*, *Anas platyrhynchos*, and *Anas poecilorhyncha*) from South Korea. Employing two rounds of pan-CoV real-time reverse transcription-polymerase chain reaction (RT-PCR) and nested PCR (nPCR) assays amplifying the conserved RNA-dependent RNA polymerase (RdRp) portion common to all known CoVs, we screened 2120 duck fecal samples collected during 2022–2023. The results indicated the presence of CoVs in 4.2% (91/2120) of samples from migratory ducks. Nucleotide sequencing of the RdRp gene revealed that all identified CoVs were clustered within the *Gamma-CoV* genus. Further phylogenetic analysis suggested that South Korean gamma-CoVs belong to the *Igacovirus* subgenus and share similarities with those found worldwide, highlighting the critical role of migratory ducks in introducing and exporting avian CoVs. We discovered two clade VII igacovirus strains in wild ducks closely related to those in pigeons, implying potential cross infection between these avian species. Overall, our study underscores the importance of active surveillance and monitoring of avian CoVs in wild birds as a preemptive response against the forthcoming emergence of new CoV species that can threaten both animal and human health.

## 1. Introduction

Coronaviruses (CoVs) constitute a highly diverse group of enveloped single-stranded, positive-sense RNA viruses, primarily responsible for causing significant respiratory and enteric diseases in humans and diverse companion, domestic, and wild animals, posing a socioeconomic threat to both humans and animals [1–4]. Characterized by the largest RNA genome (~25–32 kb), CoVs belong to the subfamily *Orthocoronavirinae* within the family *Coronaviridae* of the order *Nidovirales* [5, 6]. The *Orthocoronavirinae* subfamily encompasses four genera: *Alpha*-, *Beta*-, *Gamma*-, and *Delta-CoV* [7, 8]. Alpha-CoVs and beta-CoVs are believed to have evolved from bat CoVs, infecting mammals, including humans and bats. Conversely, gamma-CoVs and delta-CoVs likely have an avian CoV ancestor, predominantly circulating among various avian orders [2, 9]. The broad range of host species facilitates the frequent interspecies spillover of CoVs into new hosts (i.e., from animals to humans or from wild to domestic animals) [9–12].

Wild birds, along with bats serving as genetic sources for alpha- and beta-CoVs, function as natural reservoirs for diverse gamma- and delta-CoVs [13]. Migratory birds, with their ability to cover long distances between breeding and nonbreeding locations and their gregarious behaviors, can act as transmission vectors for viral dispersal and contributors to viral recombination [14]. Notably, migratory ducks belonging to the *Anseriformes* order are recognized hosts for several RNA viruses, including avian CoVs [15]. The molecular epidemiology of poultry CoVs causing significant financial losses in domestic birds, such as infectious bronchitis virus and turkey CoV, has been broadly investigated [16–19]. In contrast, limited studies have explored the evolution, genetic variation, prevalence, and geographical circulation of CoVs in wild birds, especially migratory ducks. Moreover, avian-origin gamma- and delta-CoVs have been identified in multiple mammalian species, such as cetaceans, leopard cats, and pigs, suggesting ongoing cross species transmission (CST) from birds to mammals [20, 21]. Considering this circumstance, continuous surveillance and monitoring of avian CoVs in wild bird populations are crucial to anticipate potential zoonotic transmission of viruses to livestock and humans. Hence, the present investigation aimed to ascertain the occurrence and genetic traits of CoVs in migratory ducks from South Korea.

## 2. Materials and Methods

### 2.1. Specimen Collection for Surveillance

The duck fecal samples (*n* = 2120) analyzed in this study were sourced from the National Avian Influenza Virus Surveillance Program (NAIVSP) by the Ministry of Agriculture, Food, and Rural Affairs that collected fresh feces on the ground from various locations in South Korea between 2022 and 2023. The selected duck species included the Northern pintail (*Anas acuta*), Mallard (*Anas platyrhynchos*), and spot-billed duck (*Anas poecilorhyncha*).

To process the fecal samples, they were initially prepared as 10% (wt/vol) suspensions in phosphate-buffered saline (PBS), and these suspensions then underwent vertexing and centrifugation at 4500 × *g* for 10 min (Hanil Centrifuge FLETA5, Incheon, South Korea), as described previously [22–24]. Total RNA extraction from the fecal suspension was carried out utilizing *i*-TGE/PED detection kits (iNtRON Biotechnology, Seongnam, South Korea) in accordance with the manufacturer's protocol. The extracted RNA was then stored at −80°C until further analysis.

### 2.2. Pan-CoV Real-Time Reverse Transcription-Polymerase Chain Reaction (pan-CoV rRT-PCR) and Nested PCR (nPCR)

The extracted RNA was initially screened for CoVs through a broad-spectrum pan-CoV rRT-PCR assay, amplifying the conserved part of the RNA-dependent RNA polymerase (RdRp) of CoVs, as described previously [25, 26]. The pan-CoV rRT-PCR (1^st^ round of PCR) was performed using One Step TB Green PrimeScript RT-PCR Kits (TaKaRa, Otsu, Japan) following the manufacturer's instructions. The mixtures were reacted utilizing a CronoSTAR 96 Real-Time System (Clontech, Mountain View, CA), and the results were analyzed with the system software as described elsewhere [4, 27]. Fecal specimens with cycle threshold (*Ct*) values of <39 were considered positive for CoVs. Subsequently, the CoV-positive RT-PCR products were subjected to nPCR (2^nd^ round of PCR) to amplify a 422-bp product within the first PCR, as described previously [26].

### 2.3. Nucleotide Sequencing and Phylogenetic Analysis

The final PCR amplicons of the expected size were purified using Expin Gel SV (GeneAll Biotechnology, Seoul, South Korea) according to the manufacturer's protocol and subsequently sequenced. Sequencing of the CoV RdRp gene was conducted bidirectionally with gene-specific primers from the second nPCR using the Sanger method. The acquired sequences were trimmed, and consensus nucleotides were compared with those in the GenBank database utilizing a Basic Local Alignment Search Tool (BLAST) query to identify the CoV genus. The final RdRp sequences of CoVs, denoted as GNU-DuCoV-1–78, determined in this study were deposited in GenBank under accession numbers PP027975–8052.

The RdRp sequences, along with 175 reference strains from *Alpha*- (8), *Beta*- (9), *Gamma*- (149), and *Delta-CoV* (9) genera retrieved from the GenBank database, were aligned utilizing the ClustalX 2.0 program, and the percentages of nucleotide sequence divergences were evaluated using the same software, as described previously [28]. Phylogenetic trees were created from the aligned nucleotide sequences using the neighbor-joining method with 1000 bootstrap replicates, employing Mega X software, as described elsewhere [29, 30].

## 3. Results

### 3.1. Prevalence of Gamma-CoVs in Anseriformes

In the mainland of South Korea, surveillance conducted from 2022 to 2023 involved the examination of 2120 duck samples from three *Anseriformes* species. The initial pan-CoV rRT-PCR analysis revealed the presence of CoVs in 91 out of the 2120 samples tested (4.2%). Positive samples were identified across three duck species: northern pintail (31), mallard (28), and Indian spot-billed duck (32).

Among the 91 CoV-positive samples, 78 exhibited amplicons of the expected size (85.7%; 95% confidence interval (CI): 77.1%–91.5%) and underwent sequencing to determine the CoV genus. Bidirectional sequencing of these 78 samples, coupled with nucleotide BLAST analysis, confirmed their classification as *Gamma-CoV*. Mallards and Indian spot-billed ducks displayed a higher prevalence, with 28 (35.9%) and 27 (34.6%) of the 78 samples testing positive, respectively, while northern pintails showed less viropositivity (29.5%). Sequence analysis within the obtained *gamma-CoV* strains (GNU-DuCoV-1–78) revealed identity ranges of 82.5%–100% among themselves, and they exhibited the highest homology (83.5%–100%) to sequences from duck *gamma-CoVs* identified globally (Table 1 and Supporting Information Table S1).

### 3.2. Phylogenetic Analyses of *Gamma-CoVs* in Wild Birds

**Figure 1:**
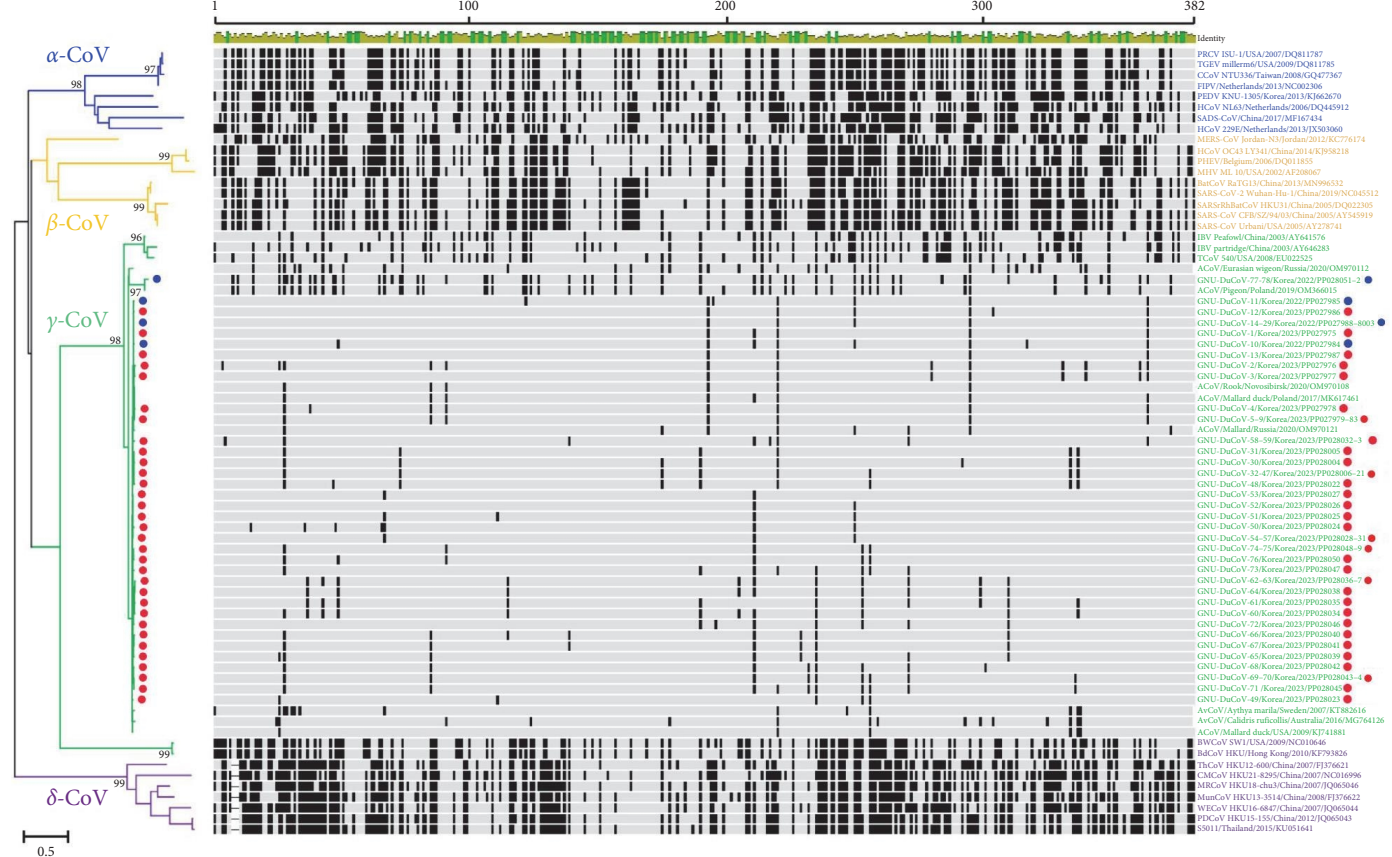
Phylogenetic analysis and barcode profiles based on RdRp gene sequences from four CoV genera (*Alpha*-*CoV*, *Beta*-*CoV*, *Gamma*-*CoV*, and *Delta*-*CoV*). Numbers at each branch represent bootstrap values exceeding 50%, determined from 1000 replicates. The four CoV genera, *α*-*CoV* (blue), *β*-*CoV* (orange), *γ*-*CoV* (green), and *δ*-*CoV* (purple) are indicated. Different colored circles on the green (*γ*-*CoV*) branches indicate *gamma-CoVs* identified chronologically in this study: blue circles for 2022 strains and red circles for 2023 strains. The scale bar indicates nucleotide substitutions per site. A schematic diagram (barcode profiles) of the CoV RdRp gene alignment in relation to the overall consensus sequence derived from at least 50% of the RdRp sequences of the 115 CoV species, was generated using Geneious software version 2023.2.1. Lightly shaded regions depict similarity to the consensus nucleotide sequence, while black bars represent mutations from the consensus sequence. Thin horizontal dashed lines indicate deleted nucleotides. The name, isolation country and date (year), and GenBank accession number of each CoV strain are presented on the right. CoV, coronavirus; RdRp, RNA-dependent RNA polymerase.

**Figure 2:**
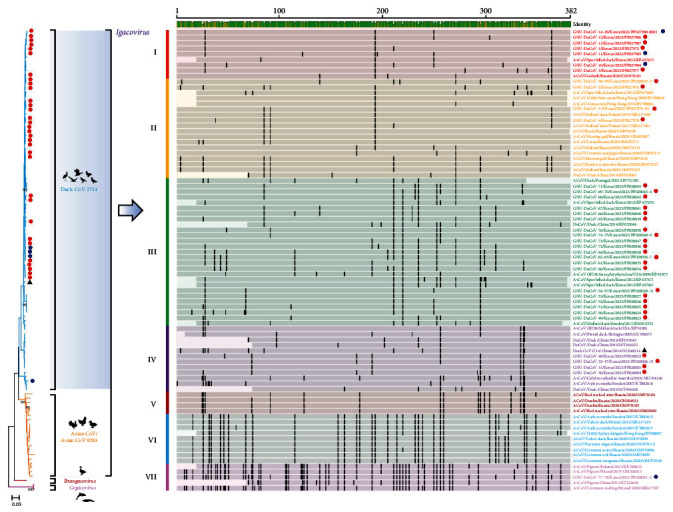
Phylogenetic analysis and barcode profiles based on RdRp gene sequences of the *GammaCoV* genus. Numbers at each branch are bootstrap values exceeding 50%, determined from 1000 replicates. Three gamma-CoV subgenera, *Igacovirus* (two groups, duck-CoV-2714 [sky blue] and *Avian CoV*/*Avian CoV 9203* [dark orange]), *Brangacovirus* (dark red), and *Cegacovirus* (pink), are indicated with corresponding host silhouette images. Blue (2022) and red (2023) circles on the sky blue (the duck-CoV-2714 group in the *Igacovirus* subgenus) branches indicate the duck igacovirus strains identified chronologically in this study. A black triangle signifies a reference stain of the duck-CoV-2714 group in the *Igacovirus* subgenus. The scale bar indicates nucleotide substitutions per site. A schematic diagram (barcode profiles) of the *igacovirus* RdRp gene alignment, in relation to the overall consensus sequence derived from at least 50% of the RdRp sequences of the 126 *igacovirus* strains, was generated using Geneious software version 2023.2.1. Lightly shaded regions indicate similarity to the consensus nucleotide sequence, while vertical black bars represent mutations from the consensus sequence. Thin horizontal dashed lines indicate deleted nucleotides. *Igacoviruses* were clustered into seven clades, and individual clades are shaded in different colors as follows: red (clade I), orange (clade II), green (clade III), purple (clade IV), dark red (clade V), sky blue (clade VI), and pink (clade VII). The name, isolation country and date (year), and GenBank accession number of each CoV strain are presented on the right. CoV, coronavirus; RdRp, RNA-dependent RNA polymerase.

Subsequently, we conducted phylogenetic analyses using the obtained RdRp gene sequences of the GNU-DuCoV-1–78 strains and those available from the GenBank database, along with representative CoV sequences from four genera. The RdRp sequence-based phylogeny confirmed the grouping of all GNU-DuCoV within the *Gamma-CoVs* genus. The barcode patterns, each representing a distinct locus from the consensus sequence, were clearly distinct from other barcode patterns representing each genus (Figure 1). A more detailed phylogenetic tree, generated on the basis of the RdRp sequences of the 78 GNU-DuCoVs and 71 reference *gamma-CoV* strains found globally, revealed three subgenera, *Igacovirus*, *Brangacovirus*, and *Cegacovirus* (Figure 2). Within the *Igacovirus* subgenus, two groups emerged (duck-CoV-2714 and Avian CoV/Avian CoV 9203). The phylogenetic analysis of the GNU-DuCoVs detected in the present study demonstrated that all 78 viruses belong to the *Igacovirus* subgenus and are further sorted into the duck-CoV-2714 group.

Using barcode profiles, *igacoviruses* within the *Gamma-CoV* genus were further clustered into seven distinct clades (I–VII; Figure 2). Detailed information on *igacovirus* clades, including the number of positive samples, geographical distribution, and host species, is presented in Table 2. The majority of GNU-DuCoV strains (76/78) were distributed among clades I–IV. None of the South Korean duck *gamma-CoVs* identified in this study belonged to clades V and VI. Interestingly, two duck *gamma-CoVs* (GNU-DuCoV-77 and −78) were organized into clade VII, commonly found in pigeon species from other countries [31–34]. This suggests a notable genetic relatedness between duck- and pigeon-dominant gamma-CoVs.

## 4. Discussion

The rise of highly contagious and pathogenic CoVs causing severe respiratory or enteric infections in humans and animals emphasizes the critical significance of understanding CoV origins and host switching [4, 35]. Despite having a broad host range spanning from mammals to birds, CoVs consistently breach host species barriers, expanding their range and posing a risk of zoonotic emergence that endangers both human and animal health [35]. Notably, CoVs within the *Gamma-CoV* and *Delta-CoV* genera are omnipresent in domestic and wild birds, facilitating the spread of various CoVs to other avian or mammalian species [13]. The emergence of mammalian delta-CoVs, such as porcine deltaCoV (PDCoV), causing mild to fatal enteritis in newborn piglets, indicates frequent CoV spillover incidents from avian to mammalian reservoirs [36]. Consequently, there is a growing research interest in understanding the presence of CoVs in wild birds. In the present study, we conducted molecular identification and genetic classification of CoVs in migratory ducks from South Korea.

In South Korea, a prior study reported a low prevalence of virus carriers among wild birds [37]. This report identified northern pintails (*A. acuta*) and Indian spot-billed ducks (*A. poecilorhyncha*), both belonging to the *Anseriformes* order, as the most prominent hosts in South Korea. None of the other avian orders, including the order *Charadriiformes* (gulls, terns, and shorebirds), harbored CoVs [37]. The detection percentage of CoVs among the three *Anas* species ranged from 3.7% to 4.2%, depending on the initial and subsequent screening. This rate is higher than the 0.95% detection rate reported in a previous study [37]. However, the prevalence of CoV among wildfowl in the *Anseriformes* order is relatively lower compared to screening data from other countries, where considerably higher CoV prevalence values were observed. Surveillance studies in China and Australia reported prevalence rates of 8.4% and 26.4% among the *Anseriformes* order, respectively [25, 38]. Independent investigations in European countries reported varying percentages of virus carriers, reaching 13.9% in Russia [26], 20% in Norway [39], 12.0%–20.5% in Sweden [40, 41], and 33.3% in Portugal [42]. These differences in prevalence rates might result from factors such as the number of bird samples, the method and geographical location of sampling, and/or the sensitivity of RT-PCR.

Similar to a previous report in South Korea, all CoVs identified in the present study were characterized as belonging to the *Igacovirus* subgenus within the *Gamma-CoV* genus. Although our sampled duck species did not show any circulation of *delta-CoVs*, it is noteworthy that the detection rate of *delta-CoVs* in aquatic birds from the *Anseriformes* order is typically lower than that of *gamma-CoVs* [25, 26, 38]. Given that both CoV genera have been reported to cocirculate among waterfowl in other countries [25, 26], the very low prevalence of *delta-CoVs* may be a circumstance in aquatic birds from South Korea. The absence of duck hosts actively shedding *alpha-CoVs* or *beta-CoVs*, as confirmed in our study, aligns with previously published surveillance data from other countries [13, 26, 43]. This leads us to hypothesize that mammalian species may serve as exclusive reservoirs and hosts for *alpha-CoVs* and *beta-CoVs*, or alternatively, these CoVs have not yet been detected in avian species. Considering the potential of any CoVs to cross species barriers and pose a threat to humans [26, 44], further investigations are essential to verify the presence of such CoVs among domestic and wild birds. Additionally, the incidence of coinfection with CoV and influenza virus has been reported in wildfowl, mainly from *Anseriformes* [38]. Although our study cannot provide insights into this aspect due to the use of NAIVSP samples negative for influenza virus, further surveillance studies will be conducted to monitor the occurrence of coinfection with these two types of viruses in wild birds from South Korea.

Our phylogenetic analysis revealed that all circulating *gamma-CoVs* in wild ducks from South Korea belonged to the *Igacovirus* subgenus, further subdivided into the duck-CoV-2714 group, displaying genetic proximity to strains found worldwide. This outcome suggests that these aquatic ducks play a crucial role as vectors, facilitating the movement of avian CoVs into and out of South Korea. *Igacovirus* strains within the *Gamma-CoV* genus were clustered into seven clades, with the majority of CoVs shed by wild ducks falling into clades I–IV. Notably, two duck *igacovirus* strains identified in this study were assigned to clade VII, a category almost exclusively comprising pigeon strains. This marks the first report of clade VII *igacovirus* in the country. These two clade VII viruses, obtained from wild ducks, exhibited a close genetic relationship with Chinese, Finnish, Polish, and Spanish pigeon *igacovirus* strains [31–34]. The high genetic similarity (95.2%–97.4%) observed between *igacoviruses* predominantly found in aquatic ducks and those dominant in pigeons suggests the potential for CST of avian CoVs between these two host species. Pigeons, known for their adaptability to urban life, pigeon racing, and varied food sources, are ubiquitous worldwide, including in South Korea [32, 45, 46]. Although our data do not provide an explanation for how CST of CoV emerges between two host species, increasing evidence suggests that contact between pigeons and wild ducks during free flights might be a possible scenario, thereby triggering cross infection between these flying birds [33, 34].

As CoVs persistently evolve, breach host species barriers, and broaden their host range [35], wildlife, especially aquatic birds, is subject to ongoing epidemiological scrutiny to understand their potential roles as natural hosts and/or reservoirs for emerging CoVs, which could pose a phantom menace to wildlife and humans [42]. Our surveillance showed the presence of *gamma-CoVs* in wild ducks, highlighting their capability to harbor and transmit CoVs. This finding further emphasizes the critical need for continuous surveillance and monitoring of CoVs in wild duck populations. Such efforts are essential for comprehending the evolution of these viruses, tracking the migration patterns of wild ducks in close vicinity to livestock and human populations, and proactively hunting emerging CoVs that could potentially lead to spillover events affecting poultry, livestock, and humans.

## 5. Conclusions

Our molecular-based surveillance study revealed the presence of *igacoviruses* from the *Gamma-CoV* genus circulating in migratory ducks from South Korea. Additionally, we identified a pigeon-dominant *igacovirus* in duck species. Avian CoVs have attracted considerable attention from the poultry and livestock industries and the research community because of their spillover ability and potential antigenic shift (i.e., recombination) capacity that can lead to the emergence of new variants. Thus, incessant efforts are needed toward active surveillance and monitoring of avian pathogenic CoVs, along with vigilance for emerging new CoVs in birds and livestock, to preemptively respond to potential threats to both animal and human health.

## Figures and Tables

**Table 1:** Comparison of RdRp gene sequences of the South Korean duck *gamma-CoVs* identified in this study to the global duck gammacoronavirues.

Strain	Nucleotide identity (%; No. of nucleotide differences)							
	OM970101^a^	MK617448	OM970103	KJ741873	KJ741881	NC048214	KT882616	MK617419
GNU-DuCoV-1	90.8 (35)	99.0 (4)	97.6 (9)	97.1 (11)	97.6 (9)	96.9 (12)	95.5 (17)	90.8 (35)
GNU-DuCoV-2	90.3 (37)	98.4 (6)	97.6 (9)	95.0 (19)	96.1 (15)	95.8 (16)	94.5 (21)	90.3 (37)
GNU-DuCoV-3	90.6 (36)	98.2 (7)	96.3 (14)	95.8 (16)	96.9 (12)	95.5 (17)	94.8 (20)	90.6 (36)
GNU-DuCoV-4	90.6 (36)	99.7 (1)	97.9 (8)	96.3 (14)	96.9 (12)	96.1 (15)	95.3 (18)	90.6 (36)
GNU-DuCoV-5–9	90.8 (35)	100 (0)	98.2 (7)	96.6 (13)	97.1 (11)	96.3 (14)	95.5 (17)	90.8 (35)
GNU-DuCoV-10	90.6 (36)	98.2 (7)	96.9 (12)	96.3 (14)	96.9 (12)	96.1 (15)	94.8 (20)	90.6 (36)
GNU-DuCoV-11	90.8 (35)	98.4 (6)	96.6 (13)	96.1 (15)	97.1 (11)	95.8 (16)	95.0 (19)	90.8 (35)
GNU-DuCoV-12	91.6 (32)	98.7 (5)	96.9 (12)	96.3 (14)	97.4 (10)	96.1 (15)	95.3 (18)	91.6 (32)
GNU-DuCoV-13	91.1 (34)	99.2 (3)	97.4 (10)	96.9 (12)	97.9 (8)	96.6 (13)	95.8 (16)	91.1 (34)
GNU-DuCoV-14–29	91.4 (33)	99.0 (4)	97.1 (11)	96.6 (13)	97.6 (9)	96.3 (14)	95.5 (17)	91.4 (33)
GNU-DuCoV-30	90.3 (37)	96.9 (12)	96.3 (14)	96.1 (15)	97.9 (8)	96.1 (15)	96.3 (14)	90.3 (37)
GNU-DuCoV-31	90.6 (36)	97.6 (9)	96.9 (12)	96.9 (12)	98.4 (6)	96.6 (13)	96.9 (12)	90.6 (36)
GNU-DuCoV-32–47	90.8 (35)	97.1 (11)	96.3 (14)	96.6 (13)	98.4 (6)	96.3 (14)	96.9 (12)	90.8 (35)
GNU-DuCoV-48	90.6 (36)	96.9 (12)	96.1 (15)	96.3 (14)	98.2 (7)	96.1 (15)	96.6 (13)	90.6 (36)
GNU-DuCoV-49	91.1 (34)	96.9 (12)	96.6 (13)	97.1 (11)	97.9 (8)	96.1 (15)	95.8 (16)	91.1 (34)
GNU-DuCoV-50	90.1 (38)	96.3 (14)	96.1 (15)	96.6 (13)	96.6 (13)	95.3 (18)	95.0 (19)	90.1 (38)
GNU-DuCoV-51	90.8 (35)	96.9 (12)	96.9 (12)	96.6 (13)	97.4 (10)	96.1 (15)	95.8 (16)	90.8 (35)
GNU-DuCoV-52	91.1 (34)	97.4 (10)	97.2 (11)	97.1 (11)	97.6 (9)	96.3 (14)	95.8 (16)	91.1 (34)
GNU-DuCoV-53	90.8 (35)	97.6 (9)	97.4 (10)	97.4 (10)	97.9 (8)	96.6 (13)	96.3 (14)	90.8 (35)
GNU-DuCoV-54–57	91.1 (34)	97.4 (10)	97.1 (11)	97.1 (11)	97.6 (9)	96.3 (14)	96.1 (15)	91.1 (34)
GNU-DuCoV-58–59	89.5 (40)	97.6 (9)	97.9 (8)	96.3 (14)	96.9 (12)	95.5 (17)	95.3 (18)	89.5 (40)
GNU-DuCoV-60	91.6 (32)	95.5 (17)	95.3 (18)	96.9 (12)	95.8 (16)	94.5 (21)	94.2 (22)	91.6 (32)
GNU-DuCoV-61	90.8 (35)	95.3 (18)	95.0 (19)	96.1 (15)	96.1 (15)	94.2 (22)	94.0 (23)	90.8 (35)
GNU-DuCoV-62–63	90.8 (35)	95.3 (18)	95.5 (17)	96.1 (15)	95.5 (17)	94.2 (22)	93.5 (25)	90.8 (35)
GNU-DuCoV-64	90.6 (36)	95.5 (17)	95.8 (16)	96.3 (14)	95.8 (16)	94.5 (21)	93.7 (24)	90.6 (36)
GNU-DuCoV-65	91.1 (34)	97.1 (11)	97.9 (8)	96.9 (12)	96.9 (12)	96.1 (15)	95.3 (18)	91.1 (34)
GNU-DuCoV-66	91.1 (34)	97.1 (11)	96.9 (12)	97.4 (10)	96.3 (14)	95.5 (17)	94.8 (20)	91.1 (34)
GNU-DuCoV-67	90.8 (35)	97.4 (10)	97.1 (11)	97.1 (11)	96.6 (13)	95.8 (16)	95.0 (19)	90.8 (35)
GNU-DuCoV-68	90.3 (37)	97.1 (11)	97.4 (10)	96.9 (12)	96.3 (14)	95.5 (17)	94.8 (20)	90.3 (37)
GNU-DuCoV-69–70	90.3 (37)	96.9 (12)	97.1 (11)	97.1 (11)	96.3 (14)	95.5 (17)	94.8 (20)	90.3 (37)
GNU-DuCoV-71	90.1 (38)	97.1 (11)	97.4 (10)	96.9 (12)	96.6 (13)	95.8 (16)	95.0 (19)	90.1 (38)
GNU-DuCoV-72	90.8 (35)	96.3 (14)	96.6 (13)	97.1 (11)	96.6 (13)	95.3 (18)	94.5 (21)	90.8 (35)
GNU-DuCoV-73	90.6 (36)	96.9 (12)	97.1 (11)	97.6 (9)	96.9 (12)	95.5 (17)	95.3 (18)	90.6 (36)
GNU-DuCoV-74–75	90.6 (36)	97.9 (8)	97.6 (9)	97.6 (9)	97.4 (10)	96.1 (15)	95.8 (16)	90.6 (36)
GNU-DuCoV-76	90.3 (37)	97.9 (9)	97.4 (10)	97.4 (10)	97.1 (11)	95.8 (16)	95.5 (17)	90.3 (37)
GNU-DuCoV-77–78	85.9 (54)	84.6 (59)	84.6 (61)	85.3 (50)	83.8 (62)	83.5 (63)	83.5 (63)	85.9 (54)

Abbreviations: CoV, coronaviruse; RdRp, RNA-dependent RNA polymerase.

^a^GenBank accession number.

**Table 2:** An overview of the *igacovirus* clades proposed in this study.

Clade	No. positive samples	Geographical distribution	Host species
I	22	South Korea and Russia	Spot-billed duck and gadwall
II	9	South Korea, China, Hong Kong, Poland, Russia, and Bangladesh	Northern pintail, mallard, spot-billed duck, northern shoveler, herring gull, common sandpiper, rook, and mute swan
III	26	South Korea, China, USA, Sweden, Portugal, Bangladesh, and Poland	Northern pintail, mallard, spot-billed duck, northern shoveler, gadwall, and swan goose
IV	19	South Korea, China, USA, Beringia, Sweden, and Hong Kong	Mallard, northern pintail, and greater scaup
V	0	Australia and Russia	Dunlin and red-necked stint
VI	0	Hong Kong, Russia, Sweden, and Poland	Tufted duck, Eurasian wigeon, common scoter, common teal, common merganser, and greater scaup
VII	2	South Korea, China, Finland, Poland, and Spain	Pigeon, mallard, and common starling

## Data Availability

The data that support the findings of the study are included within the article, and the obtained RdRp sequences were deposited in the GenBank database.

## Supporting Information

Supporting Information Table S1: nucleotide homology of clade-specific RdRp gene sequences of *igacoviruses*.
