# Supplementary material for: Incidence and Genetic Investigation of Avian Coronaviruses in Migratory Ducks From South Korea
Source: Transbound Emerg Dis. 2024 Nov 1;2024:9502737. doi: 10.1155/2024/9502737 (PMC12016717; doi:10.1155/2024/9502737)
Supplement: Supporting Information — Table S1: nucleotide homology of clade-specific RdRp gene sequences of igacoviruses. [file 9502737.f1.pdf]

TABLE S1: Nucleotide homology of clade-specific RdRp gene sequences of igacoviruses.

Clade	Nucleotide identity (%) (No. of nucleotide differences)	
	Identity among the GNU-DuCoVs	Identity to the global strains
I	97.9–99.7 (1–8)	97.9–99.4 (2–8)
II	96.1–99.7 (1–15)	96.3–100 (0–14)
III	95.3–99.7 (1–18)	95.8–98.7 (5–16)
IV	99.0–99.7 (1–4)	96.1–98.4 (6–15)
V	-[a]	-
VI	-	-
VII	100 (0)	95.5–97.4 (10–17)

Note. [a]None of the GNU-DuCoVs identified in this study belonged to clades V and VI.
